# Prevalence of intestinal parasitic infections in Hawassa University students’ clinic, Southern Ethiopia: a 10-year retrospective study

**DOI:** 10.1186/s13104-019-4747-5

**Published:** 2019-10-28

**Authors:** Tadesse Menjetta, Teketel Simion, Wondimu Anjulo, Kidist Ayele, Mekides Haile, Tewodros Tafesse, Solomon Asnake

**Affiliations:** 1 Department of Medical Laboratory, Hawassa University College of Health Sciences, P.O. Box 1560, Hawassa, Ethiopia; 2 Hawassa Health Center, Hawassa, Ethiopia

**Keywords:** Parasitic infections, Helminths, Protozoa, Prevalence, Hawassa University

## Abstract

**Objective:**

The purpose of this study was to determine the prevalence of intestinal parasitic infections among patients who had attended Hawassa University students’ clinic, Southern Ethiopia.

**Result:**

Over the 10 years period, a total of 13,679 patients visiting Hawassa University students’ clinic were included in the study. A total of 6553 (47.9%) patients were positive for at least one intestinal parasite. The overall prevalence of intestinal helminth and protozoan infections was 20.3% and 27.6% respectively. There were four dual infections and one triple infection. *E. histolytica/E. dispar trophozoite* was the most common identified parasite, which was seen in 18% of the patients while *Enterobius vermicularis* (0.1%) was the least reported parasite. Other parasites identified were *Ascaris lumbricoides* (15.0%), Hookworm species (2.0%), Taenia species (1.8%), *Hymenolepis nana* (0.7%), *Strongyloides stercoralis* (0.3%), *Trichuris trichuria* (0.2%), and *Shistosoma mansoni* (0.2%). The prevalence of helminthes was higher in females (23.3%) than in males (19.5%) (P < 0.00001) while that of protozoan infections was 28.5% in males than females (23.8%) (574/2414) (P < 0.00001).

## Introduction

Human intestinal parasitic infections are still the major causes of sickness and death henceforth, significant general medical issue globally [1]. It is assessed that 3.5 billion individuals are influenced world wide and 450 million are sick because of these infectivity. Regardless of whether they have an overall appropriation, they are increasingly predominant in developing countries causing real general medical issues [2].

In sub-Saharan Africa, the prevalence of intestinal parasitic diseases is high, and its rate can extend up to 95%. In these counties up to 250 million individuals are assessed to be infected with something like at least one type of intestinal nematodes [3]. These parasitic diseases are brought about by both protozoa and helminthes parasites [4].

The study of disease transmission of intestinal parasitic infections demonstrates that these parasites are found in each age gathering and in both genders. Be that as it may, the rate is distinctive in certain areas and in some age groups [5]. Studies completed in various nations have demonstrated that the circumstance of an individual is a significant reason in the predominance of intestinal parasitic contamination [6]. High predominance is found in individuals with low financial status, poor living condition, stuffed regions, poor ecological sanitation, inappropriate trash transfer, hazardous water supply and unhygienic individual habits. These components are the reason for real extent of weight of the infection and death in developing countries [7]. In Ethiopia, few investigations were done among various societies to demonstrate the predominance of intestinal parasitosis. It could be exceptionally persistent due to poor financial measures, poor ecological sanitation and unawareness of components like hand washing, utilization of restroom and utilization of crude/half-cooked vegetables or dairy and meat items [8]. Even if there are investigations directed to survey the circumstance of intestinal parasitosis in various pieces of Ethiopia, there are yet regions for which epidemiological data isn’t accessible including the present study area. Therefore, this retrospective study is aimed to assess the prevalence of intestinal parasitosis among patients in Hawassa University students’ clinic, Southern Ethiopia.

## Main text

### Methods

#### Study design and period

This 10 years retrospective study was conducted in Hawassa city, Southern Ethiopia from August 2008 to July 2018.

#### Study area

Hawassa city is located 273 km away from Addis Ababa capital of Ethiopia. The city has total population of 367,970. It is surrounded by Lake Hawassa. The city has an area of 157.2 SQ km.

#### Study population

All Patients who had been examined for stool sample and having complete age, sex, and stool examination documentation over the study period.

#### Sample size determination

All patients’ data with complete age, sex, and stool examination documentation over the aforementioned study period were included in this retrospective study.

#### Data collection and data quality control

Using data extraction sheet/form, all required information for this retrospective study was obtained from the registration/record books of the Hawassa University students’ clinic. After data collection process, the data was rechecked and cleaned.

#### Data analysis plan

All data were double analyzed using SPSS (version 23.0) statistical packages. Descriptive statistics were used for analysis. Chi square test (χ^2^) was used to determine association between prevalence by gender and age. *P* value was considered to be statistically significant when *P* value is less than 0.05. Additionally tables were used to display the results.

#### Ethical consideration

Ethical clearance was obtained from research and ethics review committee of Hawassa University. Official permission was also obtained from clinic administrators.

### Results

From a total of 13,679 patients who visited Hawassa University students’ clinic laboratory for stool examination having a complete age, sex and stool examination results, 6553 patients were found to be positive for intestinal parasite making the overall prevalence of parasitic infections 47.9%. There were nine different parasites encountered. The most common intestinal parasites identified were *Entamoeba histolytica/dispar* (18.0%), *Ascaris lumbricoides* (15.0%), and *Giardia lamblia* (9.6%). *Enterobius vermicularis* showed the least prevalence (0.1%). The other parasites identified were Hookworm Species*, Trichuris trichiura, Schistosoma mansoni, Taenia* species*, Hymenolepis nana, Strongyloides stercoralis* (Table 1).

**Table 1:** Prevalence (%) of intestinal parasites stratified by year

Parasite	Year 1 No. (%)	Year 2 No. (%)	Year 3 No. (%)	Year 4 No. (%)	Year 5 No. (%)	Year 6 No. (%)	Year 7 No. (%)	Year 8 No. (%)	Year 9 No. (%)	Year 10 No. (%)	Overall prevalence
Hw	26 (2.1)	14 (1.1)	89 (5.5)	44 (2.4)	27 (2.2)	31 (2.2)	7 (0.8)	19 (1.2)	5 (0.3)	8 (0.7)	270 (2.0)
Al	26 (2.1)	35 (2.9)	125 (7.7)	240 (13.2)	155 (12.4)	177 (12.3)	96 (11.1)	457 (30.0)	327 (22.3)	408 (34.5)	2046 (15.0)
Ev	3 (0.2)	0 (0.0)	1 (0.1)	1 (0.1)	0 (0.0)	2 (0.1)	0 (0.0)	2 (0.1)	4 (0.3)	2 (0.2)	15 (0.1)
Tt	2 (0.2)	1 (0.1)	10 (0.6)	2 (0.1)	5 (0.4)	4 (0.3)	2 (0.2)	2 (0.1)	4 (0.3)	0 (0.0)	32 (0.2)
Sm	9 (0.7)	7 (0.6)	10 (0.6)	2 (0.1)	1 (0.1)	1 (0.1)	1 (0.1)	0 (0.0)	0 (0.0)	1 (0.1)	32 (0.2)
Ts	7 (0.6)	17 (1.4)	22 (1.3)	25 (1.4)	7 (0.6)	20 (1.4)	12 (1.4)	45 (3.0)	43 (2.9)	43 (3.6)	241 (1.8)
Hn	11 (0.9)	11 (0.9)	36 (2.2)	11 (0.6)	5 (0.4)	10 (0.7)	5 (0.6)	4 (0.3)	2 (0.1)	5 (0.4)	100 (0.7)
Ss	2 (0.2)	10 (0.8)	12 (0.7)	5 (0.3)	1 (0.1)	2 (0.1)	2 (0.2)	1 (0.1)	0 (0.0)	0 (0.0)	35 (0.3)
Overall prevalence of helminthes											2771 (20.3)
Eh/d	267 (21.1)	299 (24.4)	392 (24.0)	287 (15.8)	203 (16.3)	284 (19.7)	240 (27.7)	208 (13.7)	122 (8.3)	162 (13.7)	2464 (18.0)
Gl	97 (7.6)	89 (7.3)	318 (19.5)	244 (13.5)	136 (10.9)	146 (10.1)	51 (5.9)	74 (4.9)	98 (6.7)	65 (5.5)	1318 (9.6)
Overall protozoan infections											3782 (27.6)
Prevalence in each year	450 (35.5)	483 (39.4)	1015 (62.3)	861 (47.5)	540 (43.3)	677 (47.0)	416 (48.0)	812 (53.4)	605 (41.2)	694 (58.8)	6553 (47.9)
Total Sample	1268	1227	1630	1813	1246	1439	867	1522	1468	1181	13,679

Hw, Hookworm Species; Al, *Ascaris lumbricoides*; Ev, *Enterobius vermicularis*; Tt, *Trichuris trichiura*; Sm, *Schistosoma mansoni*; *Ts*, *Taenia* species; Hn, *Hymenolepis nana*; Ss, *Strongyloides stercoralis*; *Eh/d*, *Entamoeba histolytica/dispar*; *Gl*, *Giardia lamblia*

From dual IPIs *Entamoeba histolytica/dispar* and *Giardia lamblia* were most common 118 (0.7%). The second dual IPIs was *Ascaris lumbricoides* and *Entamoeba histolytica/dispar* 79 (0.6%) following Hookworm species and *Ascaris lumbricoides* 49 (0.4%). The only triple IPIs was the combination of *Entamoeba histolytica/dispar, Ascaris lumbricoides* and *Giardia lamblia* 10 (0.1%) (Table 2).

**Table 2:** Distribution (in percent) of intestinal parasites with multiple infections

Multiple parasites observed	Male (n = 11,265) No (%)	Female (n = 2414) No (%)	Total (n = 13,679) No (%)
*A. lumbricoides, E. histolytica/dispa* and *G. lamblia*	9 (0.1)	1 (0.1)	10 (0.1)
*E. histolytica/dispa* and *G. lamblia*	95 (0.8)	23 (0.9)	118 (0.7)
*A. lumbricoides,* and *E. histolytica/dispa*	61 (0.5)	18 (0.8)	79 (0.6)
*A. lumbricoides* and Hookworm species	38 (0.3)	11 (0.5)	49 (0.4)

### Discussions

Helminthic and protozoan infections specially are major health problem in Ethiopia. Many factors like, poor hygienic habits, poor standard of living, lack of health education, ignorance, poverty, poor socio-economic conditions are some of the many reasons behind high prevalence of parasitic infections. During the present study, protozoan infection was recorded to be higher compared to helminth infection. This condition usually contributed by different level of environmental conditions which facilitate the transmission of the infective stages of the parasites.

The overall prevalence rate of intestinal parasitic diseases among patients record that had complete sex, age and stool examination at Hawassa University students’ clinic, from August 2008 to July 2018 was 47.9%. Such a rate of parasitic diseases recorded in this review study could for the most part be related to the low financial condition, described by deficient water supply, poor sterile transfer of excrement, the tropical atmosphere, and absence of information about parasite transmission common of many countries, for example, those in Africa [2].

The finding of this study is lower than the findings from Nigeria (72%), Tanzania (57.1%) and Rwanda (50.5) [9, 10, 15]. This variety is likely because of contrast in time, place, method used, health alertness, and living values. However it is higher than the finding from Tikur Anbessa University Hospital, Ethiopia, (34.5%), Nigeria (44.5%), Wonago Health Center, Southern Ethiopia (16.6%), Senegal (23.1%) [2, 11–13].

The predominant parasite identified in the present study was *Entamoeba histolytica/dispar* with a prevalence of (18%). This was higher than the findings of studies conducted in Tikur Anbessa University Hospital, Ethiopia (13.6%) and Wonago Health Center, Southern Ethiopia (8.9%]) [2, 12]. The higher prevalence of parasites like *Entamoeba histolytica/dispar* (18%) and *Giardia lamblia* (9.6%) in this study may be associated with poor personal and environmental hygiene in addition to having feco-oral route of transmission. This higher prevalence of these two protozoan parasites goes in line with the report of WHO which pointed out these two parasites as common causes of intestinal infection throughout Ethiopia [14].

In this study the overall prevalence of intestinal helminth infections is 20.3% and it is significantly higher in females (23.3%) than males (19.5%). Higher prevalence in females (55.7%) than males (44.32%), was found a study conducted from Mwanza, Tanzania [11]. But in study conducted in Tikur Anbessa University Hospital, Ethiopia it was higher in males (50.9%) than females (49.1%) [2].

The prevalence of protozoan infections was 27.6% and it was significantly higher in males (28.5%) than in females (23.8%) (Table 3). This finding differs from the findings in Mwanza, Tanzania it was significantly higher among in females (58.1%), than in males (42.3%) [11].

**Table 3:** Prevalence (%) of intestinal parasites stratified by gender

Parasite	Males No. (%)	Females No. (%)	Prevalence No. (%)	χ^2^	P-value
Hw	231 (2.1%)	39 (1.6%)	270 (2.0%)	1.944	0.163235
Al	1593 (14.1%)	453 (18.8%)	2046 (15.0%)	33.422	< 0.00001
Ev	12 (0.1%)	3 (0.1%)	15 (0.1%)	0.057	0.811302
Tt	26 (0.2%)	6 (0.3%)	32 (0.2%)	0.027	0.869482
Sm	29 (0.3%)	3 (0.1%)	32 (0.2%)	1.510	0.219139
Ts	188 (1.7%)	53 (2.2%)	241 (1.8%)	3.186	0.074272
Hn	86 (0.8%)	14 (0.6%)	100 (0.7%)	0.922	0.33695
Ss	32 (0.3%)	3 (0.1%)	35 (0.3%)	1.989	0.158445
Prevalence of helminths	2197 (19.5%)	574 (23.8%)	2771 (20.3%)	22.492	< 0.00001
Eh/d	2077 (18.4%)	387 (16.0%)	2464 (18.0%)	7.793	0.005245
Gl	1131 (10.0%)	187 (7.7%)	1318 (9.6%)	12.010	0.000529
prevalence of protozoan	3208 (28.5%)	574 (23.8%)	3782 (27.6%)	20.949	< 0.00001
Overal prevalence	5405 (47.9)	1148 (47.6)	6553 (47.9)	0.144	0.70475
Total samples	11,265	2414	13,679		

Statistically significant at P < 0.05, χ^2^ = Chi square

Hw, Hookworm Species; Al, *Ascaris lumbricoides*; Ev, *Enterobius vermicularis*; Tt, *Trichuris trichiura*; Sm, *Schistosoma mansoni*; *Ts*, *Taenia* species; Hn, *Hymenolepis nana*; Ss, *Strongyloides stercoralis*; *Eh/d*, *Entamoeba histolytica/dispar*; *Gl*, *Giardia lamblia*

Regarding distribution of the intestinal parasites among age groups, it was almost similar in age groups 18–23 years (48%), and in age groups 24–33 years (48.5%) but lower in age groups 34+ years (39.6%). Age group specific prevalence of helminths for age group 18–23 years, 24–33 years and 34+ years were 20.3%, 20.1% and 18.9% respectively. Age group specific prevalence of protozoan for age group 18–23 years, 24–33 years and 34+ years were 27.7%, 28.4% and 20.7% respectively.

### Conclusions

The overall prevalence of intestinal parasitic infections in this retrospective study was 47.9% and was positive for at least one intestinal parasite. *Entamoeba histolytica/dispar* was the most commonly reported parasite, which was seen in 18.0% of the patients. In conclusion, this study shows that intestinal helminthiases and protozoan infections are among the common parasitic infections observed among patients presenting at Hawassa University students’ clinic. It is necessary to develop effective prevention and control strategies including health education and improving environmental sanitation.

## Limitation

In this study, since microscopic examination method was used, differentiation of *Entamoeba histolytica* from *Entamoeba dispar* in stool samples was not possible.

## Data Availability

The datasets used and/or analyzed during the current study are available from the corresponding author on reasonable request.
